# Effects of Fish Bone Meal Supplementation on Growth Performance, Blood Immunity, Intestinal Morphology, and Gut Microbiota in Laying Hens

**DOI:** 10.3390/ani15040548

**Published:** 2025-02-13

**Authors:** Kangle Wu, Fang Wang, Shihang Yang, Chongyang Zhang, Meizhu Xie, Jiayang Li, Yulong Yin, Kang Yao

**Affiliations:** 1 Institute of Subtropical Agriculture, Chinese Academy of Sciences, Changsha 410125, China; 13278881189@163.com (K.W.); wf980313@163.com (F.W.); chongyangz7931@163.com (C.Z.); yinyulong@isa.ac.cn (Y.Y.); 2 College of Animal Science and Technology, Hunan Agricultural University, Changsha 410127, Chinalijiayang0511@163.com (J.L.)

**Keywords:** poultry nutrition, soybean meal, animal protein

## Abstract

The poultry industry relies heavily on soybean meal to feed egg-laying chickens, but its high cost and price instability necessitate the exploration of more affordable and reliable alternatives. Fish bone meal, a nutritious byproduct from fish processing, offers high-quality protein and essential minerals, making it a promising addition to poultry diets. In this study, 240 Hy-Line Brown laying hens (31 weeks old, average weight 1.90 ± 0.1 kg, egg laying 11 weeks) were randomly assigned to two dietary treatments for 12 weeks: one group received a regular diet, while the other was fed a diet containing 3% fish bone meal. Each treatment included 12 replicates with 10 hens per replicate, and all birds were maintained under identical environmental conditions using a completely randomized experimental design. The results showed that hens fed the fish bone meal diet produced more eggs (*p* < 0.05) and had heavier yolks (*p* < 0.05) compared to the control group. Furthermore, intestinal health indicators, including villus height and crypt depth, were significantly improved (*p* < 0.05), along with enhanced immune markers (*p* < 0.05) in the fish bone meal group. However, no significant differences were observed in other egg quality parameters or feed conversion efficiency between the two groups (*p* > 0.05). These findings demonstrate that fish bone meal can improve egg production, yolk quality, intestinal health, and immune function in laying hens.

## Simple Summary

Soybean meal is commonly used to feed egg-laying chickens, but its high cost and unstable prices pose challenges for the poultry industry. This study explored fish bone meal, a nutritious byproduct from fish processing, as a more affordable and reliable alternative. A total of 240 Hy-Line Brown laying hens were either fed a standard diet or a diet supplemented with 3% fish bone meal for 12 weeks. The egg production, egg quality, intestinal health, and immune strength were monitored. The results showed that hens given fish bone meal laid significantly more eggs and had heavier yolks. Additionally, their intestinal health improved, with healthier gut structures, and their immune systems were stronger. There were no negative effects on other aspects of egg quality or feed efficiency. These findings suggest that fish bone meal is a cost-effective and beneficial substitute for soybean meal, providing both nutritional and health advantages. Incorporating fish bone meal can make poultry farming more sustainable by reducing reliance on traditional protein sources and effectively utilizing fish processing waste, ultimately benefiting the agricultural industry and society.

## 1. Introduction

The laying hen industry heavily depends on soybean meal as the primary protein source in feed formulations, which poses several challenges. In some countries where soybean meal is scarce, this reliance further exacerbates feed costs. Soybean meal is not only expensive but also subject to frequent price fluctuations [1]. To address the challenges posed by the high cost and price fluctuations of soybean meal, alternative protein sources have been explored. A study investigated the effects of replacing soybean meal with 5% sunflower seed meal in laying hens. The results indicated that while it improved the laying rate, there were no significant changes in the average egg weight or feed conversion ratio [2]. Similarly, research examined the impact of replacing soybean meal with 6% pea protein concentrate on the performance and egg quality of laying hens. The findings showed that these substitutes had some positive effects on the feed efficiency, but there were no significant improvements in eggshell thickness or egg protein quality [3]. While alternative protein sources like sunflower seed meal and pea protein can reduce dependency on soybean meal, they may not consistently improve all performance and egg quality parameters [4].

Animal proteins are a viable option for replacing soybean meal, including fish meal and meat and bone meal. However, these alternatives have certain limitations in practical applications. For instance, meat and bone meal is an economical alternative, but its biosecurity risks (such as pathogen transmission) restrict its use [5]. Additionally, fish meal is considered a high-quality protein source due to its rich amino acid profile and high digestibility, but its high cost limits its inclusion [6]. In contrast, fish bone meal, a by-product of fish processing, is abundant in calcium, phosphorus, and protein, which makes it potentially suitable for the nutritional needs of laying hens. Calcium and phosphorus are critical minerals for eggshell quality and skeletal health, and fish bone meal can provide sufficient mineral supplementation for laying hens while reducing feed costs. However, research on the effects of fish bone meal on production performance and egg quality in laying hens is currently very limited, and its potential needs further exploration [7,8].

This study hypothesizes that, without compromising the production performance of laying hens, the appropriate inclusion of fish bone meal in feed can effectively enhance egg quality, particularly in terms of eggshell strength and protein content. Based on this hypothesis, the study aims to systematically evaluate the effects of fish bone meal, focusing on its impact on key parameters such as egg production rate, eggshell strength, and overall egg quality. The findings will provide a scientific basis for optimizing feed formulations, improving egg quality, and promoting the high-quality development of the laying hen industry.

## 2. Materials and Methods

The study protocol was approved by the Protocol Management and Review Committee of the Institute of Subtropical Agriculture, Chinese Academy of Sciences (Approval No. 20241387). All procedures involving hens were conducted in strict accordance with the Institute of Subtropical Agriculture’s guidelines on Animal Care (Changsha, China), ensuring ethical treatment and humane slaughter.

### 2.1. Experimental Design, Birds, and Feed

A controlled henhouse environment was meticulously maintained under optimal conditions, with daily lighting scheduled from 06:00 a.m. to 22:00 p.m. Temperature and humidity were rigorously monitored at three levels—top, middle, and bottom—across the henhouse to ensure a uniform environment. Furthermore, to uphold hygiene and diminish disease risks, the henhouse was subjected to manure belt management every other day.

In this study, 240 Hy-Line Brown laying hens (31 weeks old, average weight 1.90 ± 0.1 kg) were randomly assigned to two dietary treatments: a control group (CON) and a fish bone meal group (FBP) The specific nutritional ingredients are shown in Table 1. Each dietary treatment was replicated in 12 individual cages, with ten birds housed per cage. Cages measuring 85 cm × 55 cm × 55 cm (L × W × H) were arranged in three tiers, and wooden barriers were placed between cages to prevent cross-feeding. The diets, provided in mash form, were administered under consistent environmental conditions across all groups.

The trial commenced following a 14-day adaptation period and continued for 12 weeks. The inclusion of 3% fish bone meal was based on our previous research, which found this level to be optimal for providing essential nutrients such as protein, calcium, and phosphorus, while maintaining feed intake and production performance in laying hens.

### 2.2. Laying Performance

Egg production and egg weight metrics were recorded daily to assess laying hen productivity. Concurrently, feed intake was systematically documented on a weekly basis by collecting and weighing the remaining feed to calculate feed-to-egg conversion ratios (FCR) and other critical performance indicators such as average daily feed intake, laying rate, and egg mass.

### 2.3. Egg Quality

Egg quality on study day 90 was evaluated. Two eggs from each cage were collected within a 24 h period for analyses. The measured quality parameters encompassed shell weight, shell thickness, egg shape index, shell strength, albumin height, and Haugh units. Albumin height was assessed using an automatic egg quality tester (EMT-5200, Robotmation, Changsha, China). Eggshell strength was quantified using an eggshell strength tester, which measured the force required to fracture the shell. Shell thickness was measured at three distinct points—the large end, the equatorial region, and the small end—using a thickness gauge (NFN-380, FHK, Changsha, China). Average measurements indicated overall shell thickness. The egg shape index was calculated by dividing the egg’s breadth by its length and multiplying the result by 100 to provide a quantitative assessment of the egg’s form.

### 2.4. Blood Plasma Collection and Analysis

Blood was drawn from the wing veins of selected hens (*n* = 6) into EDTA anticoagulant tubes (Shandong Aosaite Medical Devices Co., Ltd., Zibo, China). Following centrifugation at 3000× *g* for 15 min at 4 °C, separated plasma was immediately flash-frozen in liquid nitrogen and stored at −80 °C [9]. To quantify chicken-specific immune response markers—including chicken immunoglobulin A (IgA, Kit No. A-4989A 96T), chicken immunoglobulin E (IgE, Kit No. A-9423A 96T), chicken immunoglobulin G (IgG, Kit No. A-2645A 96T), chicken interleukin-10 (IL-10, Kit No. A-5023A 96T), and chicken interferon-gamma (IFN-γ, Kit No. A-2660A 96T)—plasma samples were analyzed using enzyme-linked immunosorbent assay (ELISA) (Meimian, Yancheng, China).

### 2.5. Histological Analysis of Intestines

To conduct histological analysis, jejunum samples placed in 10% formalin were sent to the histology lab for sectioning and staining [10,11]. Using a Leica Imaging System image analyzer, six images of the intestine were captured at a magnification of 200× per section. Leica LAS EZ 3.4 [12] was used for image processing and quantification. The six images per intestinal section were averaged to generate one mean value per hen. Villus height, villus width, and crypt depth were determined following established procedures, and villus surface area was estimated accordingly [13].

### 2.6. Cecal Microbial Analysis

At the end of the trial (90 days), hens were humanely culled, and their cecal contents harvested for gut microbiota analysis. Samples were immediately frozen and stored at −80 °C. For in-depth cecal microbiota examinations, we performed high-throughput sequencing, commencing with microbial genomic DNA extraction from cecal matter using QIAamp DNA Stool Mini Kits (Qiagen, Valencia, CA, USA) as per the manufacturer’s guidelines. DNA quality and quantity were examined using a NanoDrop ND1000 spectrophotometer (Thermo Fisher Scientific, Wilmington, DE, USA).

The V3 hypervariable region of the 16S rRNA gene was amplified from microbial genomic DNA using specific barcoded-fusion primers 338F (5′-ACTCCTRCGGGAGGCAGCAG-3′) and 806R (5′-GGA CTA CHVGG GTW TCTAAT-3′). PCR included an initial denaturation temperature of 94 °C for 3 min, followed by 35 cycles of denaturation, annealing, and extension steps, concluding with a final extension step. PCR products were purified from 2% agarose gels using Axyprep DNA gel extraction kits (Axygen Scientific Inc., Union City, CA, USA) and prepared for sequencing by attaching poly (A) tails and sequencing adapters. The prepared library was sequenced on an Illumina MiSeq platform by RiboBio Co., Ltd. (Changsha, China).

### 2.7. Bioinformatics Processing

Sequencing data were analyzed using the QIIME software suite (QIIME 2 2023.12.0) [14]. Initial data cleaning involved filtering out sequences based on specific criteria, such as sequence length, mismatched primers, and quality scores. After barcode and primer trimming, sequences were clustered into operational taxonomic units (OTUs) at a 97% similarity threshold to analyze gene-level composition.

### 2.8. Statistical Analyses

Data were subjected to a one-way analysis of variance (one-way ANOVA) to assess the impact of fish bone meal (FBP) supplementation compared to the control group on study outcomes. Results are presented as mean ± standard error of the mean (SEM). Prior to statistical analysis, data normality was tested using the Shapiro–Wilk test [15], and homogeneity of variances was evaluated using Levene’s test [16]. For any response variable that did not meet the normality assumption, logarithmic transformations were applied. Mean comparisons between treatments were conducted using Tukey’s honest significant difference (HSD) test to identify significant differences. Statistical analysis were performed using the Statistical Analysis System (SAS, Version 9.4, SAS Institute Inc., Cary, NC, USA) [17], with *p*-values < 0.05 considered statistically significant. For microbiota analysis, Student’s *t*-test was used to compare the relative abundance of microbial genera between the FBP and CON groups. This method was chosen because it is suitable for analyzing the differences in means between two independent groups, allowing us to identify significant changes in microbial composition at the genus level.

## 3. Results

### 3.1. Laying Performance

As shown in Table 2, In the 1–3 week period, egg weight did not differ significantly between the control group (CON) and the fish bone meal (FBP) group. The egg production rate was significantly higher in the FBP group (90.00 ± 5.55%) compared to the CON group (80.00 ± 6.23%) (*p* < 0.05) within one to three weeks. The feed conversion ratio (FCR) did not differ significantly between the CON and FBP groups. In the 4–12 week period, no significant differences were observed between the CON and FBP groups in egg weight, egg production rate, or FCR.

#### 3.1.1. Egg Quality

Table 3 shows the effects of FBP supplementation on egg quality. The FBP group had a significantly higher egg yolk weight (18.49 ± 1.01 g) than CON groups (17.16 ± 0.72 g) (*p* < 0.01). No significant differences were found between the FBP and CON groups for other egg quality parameters, including eggshell weight, thickness, aspect ratio, strength, yolk color, protein height, and Haugh unit.

#### 3.1.2. Histological Analysis of Intestines

**Figure 1:**
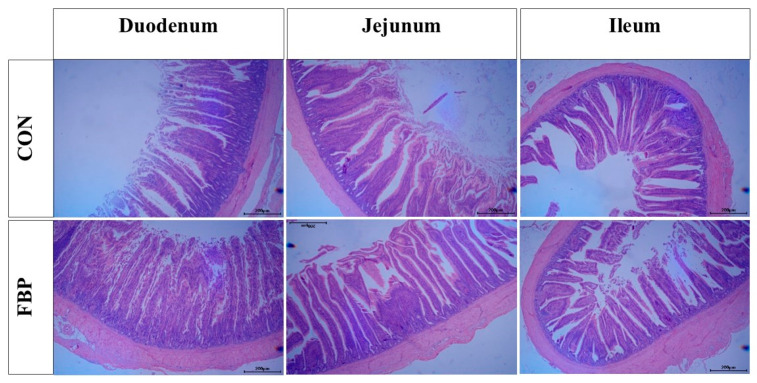
Effects of fish bone meal (FBP) supplementation compared to the basic diet (CON) on intestinal morphology in laying hens.

As shown in Table 4 and Figure 1, the FBP group had a significantly increased villus length (*p* < 0.05) and crypt depth (*p* < 0.05) in the duodenum compared to the CON group. However, no significant differences were observed in villus length or crypt depth in the jejunum and ileum (*p* > 0.05) between the two groups.

#### 3.1.3. Blood Immunity and Inflammation Indicators

As shown in Table 5, The results for blood immunity and inflammation indicators showed no significant differences in IgA and IgE levels between the CON and FBP group. However, IgG levels in the FBP group were significantly higher than those in the CON group (*p* < 0.01). Both the anti-inflammatory cytokine IL-10 and the pro-inflammatory cytokine IFN-γ were significantly elevated in the FBP group compared to the CON group (*p* < 0.01).

#### 3.1.4. Cecal Microbiota

**Figure 2:**
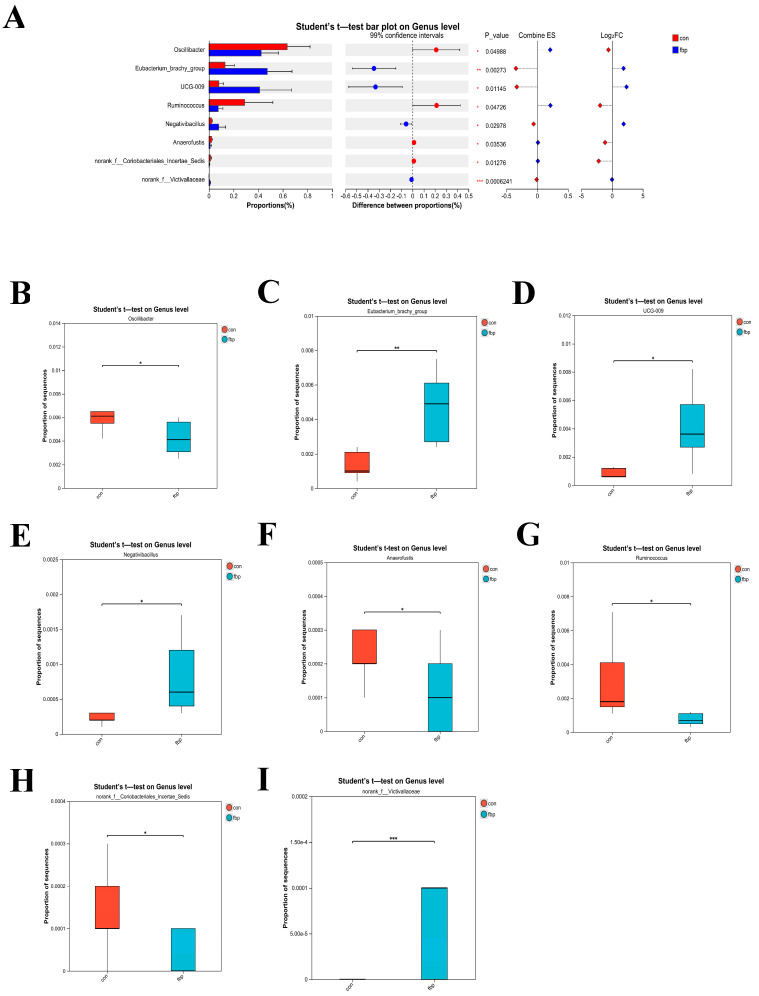
Effects of fish bone meal (FBP) supplementation compared to the basic diet (CON) on intestinal microbiota composition in laying hens. (**A**) shows the differences in the proportions of various genera between the control group (con) and treatment group (tfo) at the genus level, with 99% confidence intervals and a Student’s *t*-test. It displays *p*-Values, effect sizes (Combine ES), and Log fold change (LogFC). (**B**–**I**) show the differences in the proportions of various genera between the control group (con) and treatment group (tfo) at the gene level, with statistical analysis using a Student’s *t*-test. Statistical significance markers are as follows: * (*p* < 0.05) indicates statistical significance, ** (*p* < 0.01) indicates moderate significance, and *** (*p* < 0.001) indicates high significance.

As shown in Figure 2, the results showed that the FBP group had a significant difference in the composition of their gut microbiota. At the genus level, the abundance of *Ruminococcus* and *norank_f__Victivallaceae* was significantly higher in the fish bone meal group compared to the control group (*p* < 0.01), whereas the abundance of *Oscillibacter*, *Eubacterium_brachy_group*, *Negativibacillus*, *Anaerofustis*, and *norank_f__Coriobacteriales_Incertae_Sedis* was significantly lower in the fish bone meal group (*p* < 0.05).

## 4. Discussion

The production performance of laying hens is typically measured by parameters such as egg production rate, egg weight, and feed conversion ratio, which are key indicators of their production efficiency and directly influence the economic viability of the poultry industry [18,19]. The results demonstrated that the fish bone meal group had a significantly higher egg production rate. Previous studies have demonstrated significant differences between animal and plant protein sources in their impact on laying hens’ production performance [20]. Specifically, animal protein sources provide a more balanced profile of essential amino acids and higher digestibility, which directly impacts growth and egg-laying capacity [21]. A balanced amino acid profile refers to the presence of essential amino acids in proportions that closely match the requirements of the animal. This does not necessarily mean a higher total amount of essential amino acids, but rather the right balance to ensure optimal protein utilization and synthesis in the body. Researchers [22] have reported that supplementing the diets of aged laying hens with meat and bone meal at levels of 4% and 6% significantly improved egg production rate and eggshell quality without causing any adverse effects on the hens. Ref. [23] reported that supplementing laying hen diets with 5% fish meal for 12 weeks significantly improved egg quality and production rate. Animal protein sources are typically richer in essential amino acids, such as methionine, lysine, tryptophan, and threonine, which are relatively scarce in plant proteins, thereby limiting their nutritional value [24]. The higher egg production rate observed in the fish bone meal group may be attributed to its more balanced amino acid composition, which better meets the nutritional requirements of laying hens. However, no significant change in egg weight was observed. Egg weight is influenced by factors such as genetics, diet, and age. Factors such as the hen’s age, hormonal levels, and the availability of other key nutrients like calcium and phosphorus are also crucial in determining egg size [25]. Therefore, altering just the protein source may not be sufficient to impact egg weight significantly [26]. In terms of feed conversion ratio (FCR), the fish bone meal group showed some improvement compared to the control group; however, statistical analysis revealed that the difference was not significant [27]. Previous studies have suggested that the primary factor influencing FCR is the overall protein level in the diet rather than the composition of the protein source itself. Simply altering the protein composition may not be sufficient to effectively impact FCR [28].

Egg quality is a crucial determinant of the market value, consumer acceptance, and economic efficiency of laying hen production. In this study, the fish bone meal group exhibited a significantly higher yolk weight compared to the control group (*p* < 0.05), while other egg quality indicators remained unchanged. Researchers [29] have reported that supplementing laying hen diets with meat and bone meal had no significant effect on egg quality, which is consistent with our findings that fish bone meal did not impact most egg quality indicators. In addition, researchers [30] have noted that meat, bone meal, and plant-based proteins are digested into amino acids, which are subsequently re-synthesized for egg formation. This process implies that while various protein sources supply essential nutrients, their direct impact on egg quality is minimal, as they are eventually broken down into amino acids [31,32]. Egg quality improvement relies on the balance and efficiency of amino acid supply, particularly essential amino acids like methionine, lysine, and threonine, which are vital for protein synthesis and yolk development [33]. Yolk development begins at the early follicle stage, where the liver synthesizes yolk precursors, including vitellogenin and very-low-density lipoprotein (VLDL), which are transported to the yolk and deposited as the follicle matures [34,35]. Trace elements like zinc and selenium in fish bone meal may enhance yolk precursor synthesis and accumulation by improving follicular development and metabolic efficiency in hens [36]. These elements function as cofactors in enzymatic reactions and bolster antioxidant defenses, thus protecting follicular cells from oxidative stress [37]. This enhanced cellular environment facilitates yolk precursor synthesis and deposition, leading to a significant increase in yolk weight [38]. However, no significant effect was observed on other egg quality indicators. These findings suggest that fish bone meal improves yolk deposition by promoting follicular development and metabolic efficiency, though it has limited effects on other egg quality metrics, such as albumen height, shell thickness, and Haugh unit.

Gut health is crucial for the overall productivity and health of laying hens, as it directly affects nutrient absorption, digestion efficiency, and the hens’ ability to maintain optimal production levels [39]. Immune function becomes particularly critical during high-intensity production periods. Damage to the intestinal barrier leads to reduced nutrient absorption, redox imbalance, and impaired immune function. These factors ultimately reduce production efficiency and economic performance [40]. Researchers [41] have demonstrated that adding bone meal to poultry diets significantly improved intestinal villus length and crypt depth. Similarly, our findings show that fish bone meal led to marked improvements in these parameters. These improvements in intestinal morphology suggest that fish bone meal increases the absorptive surface area of the intestine, thereby enhancing digestive efficiency. This enhancement leads to improved nutrient uptake and overall production performance. This change is likely due to the high content of essential amino acids and trace elements in fish bone meal. These amino acids serve as the building blocks for protein synthesis. They are also crucial for the repair and regeneration of intestinal cells, particularly in maintaining epithelial cell integrity under high-stress conditions. Trace elements like zinc and selenium have antioxidant and immune-regulatory functions, maintaining intestinal barrier integrity, reducing oxidative stress-induced damage, and promoting nutrient absorption and utilization [42,43]. Researchers [44] have found that including fish bone meal in the diet significantly increased blood levels of IgG, IL-10, and IFN-γ, aligning with our results. The increase in these immune markers suggests enhanced immune protection, better regulation of inflammatory responses, and greater resilience against infections in laying hens. These increases further demonstrate the supportive effect of fish bone meal on the immune system, particularly in enhancing systemic immune defense, contributing to improved health, disease resistance, and productivity [45,46]. IgG is a key component of systemic immune defense, while IL-10 and IFN-γ are involved in anti-inflammatory and pro-inflammatory responses, respectively, playing essential roles in maintaining immune balance and defending against pathogens [47,48]. During high-intensity production, the tight junctions of the intestinal barrier in laying hens may be compromised. This disruption affects the balance of the gut microbiota, leading to the translocation of pathogens and toxins across the intestinal lumen. This leads to inflammation and tissue damage, ultimately affecting nutrient absorption and production performance [49,50]. Supplementing with fish bone meal may mitigate these negative effects by improving intestinal morphology. It also regulates the immune system, thereby enhancing the hens’ resistance to pathogens and promoting nutrient absorption [51,52]. Overall, fish bone meal as a feed additive significantly enhances gut health and immune function in laying hens by improving intestinal structure, boosting immunoglobulin levels, and regulating key cytokines such as IL-10 and IFN-γ. This regulation reduces inflammation, enhances immune balance, and ultimately improves productivity [53]. These positive effects on the overall health of the hens may also improve production performance, providing theoretical and practical support for using fish bone meal in laying hen diets.

The gut microbiota are crucial for maintaining host health, enhancing nutrient absorption, and supporting immune function. Understanding these roles is vital in the context of poultry growth, where optimizing gut health directly correlates with improved production performance [54]. These functions are essential for poultry growth and production performance [55]. In this study, fish bone meal significantly influenced the gut microbiota. This aligns with [56], who found that essential amino acids promote beneficial bacteria like *Lactobacillus* and *Bifidobacterium*, while inhibiting harmful bacteria such as *Clostridium perfringens* and *Escherichia coli*, thereby improving gut health. Fish bone meal is rich in essential amino acids, such as lysine and methionine, which are vital for supporting microbial growth and metabolism [57]. Lysine supports microbial protein synthesis, while methionine acts as a precursor for metabolites essential for microbial activity [58]. These amino acids support beneficial bacteria growth and activity while inhibiting harmful bacteria like *Clostridium perfringens* and *Escherichia coli*, thereby altering the gut microbiota composition and function [59]. This impact is evident in changes in gut bacterial genera, suggesting that fish bone meal benefits host health by modulating microbiota structure. Notably, the increase in *Ruminococcus* is significant. This genus produces butyrate—a short-chain fatty acid with anti-inflammatory properties that protects the gut barrier [60]. This indicates that fish bone meal may enhance gut health in laying hens by promoting butyrate production. *Ruminococcus* also breaks down polysaccharides into short-chain fatty acids, which are readily absorbed and used for energy, meeting the energy needs of laying hens and improving feed efficiency [61]. The reduction of harmful bacteria like *Oscillibacter* and *Eubacterium_brachy_group* may reduce negative impacts on host health, including inflammation, gut barrier dysfunction, and impaired nutrient absorption [60]. This suggests that fish bone meal may help suppress the growth of harmful microbes, thereby enhancing the proportion of beneficial bacteria and reducing gut inflammation, ultimately improving nutrient absorption and overall health. Overall, fish bone meal promotes the growth of beneficial bacteria like *Lactobacillus*, *Bifidobacterium*, and *Ruminococcus*, while inhibiting harmful bacteria such as *Clostridium perfringens* and *Escherichia coli*. This microbiota adjustment helps maintain gut balance, enhances nutrient absorption, and improves the overall health and productivity of laying hens.

## 5. Conclusions

In conclusion, this study investigated the effects of fish bone meal on laying hens, focusing on egg production, egg quality, gut health, and immune function. Our results demonstrated that fish bone meal significantly improved egg production rate and yolk quality while enhancing gut health and immune function. Fish bone meal is rich in essential amino acids, such as lysine and methionine, which are vital for supporting microbial growth and metabolism. The inclusion of fish bone meal offers a cost-effective solution to enhance productivity in regions facing soybean meal shortages. Future research should focus on determining optimal inclusion levels and assessing long-term impacts of fish bone meal. Overall, fish bone meal significantly enhances laying hens’ productivity and health, providing a viable alternative to traditional protein sources.

## Figures and Tables

**Table 1:** Composition and nutrient levels of the basic diet (CON) and the diet supplemented with fish bone meal (FBP).

Item	Control Group	FBP Rroup
Ingredient, %		
Corn (CP 8%)	60.1	61.0
Soybean meal (CP 43%)	25.60	23.00
Stone powder ^1^	9.00	9.00
Soybean oil	1	0.7
FBP ^2^	0	3
CaHPO_4_	1	
NaCl	0.3	0.3
Premix (L33) ^3^	3	3
Total	100.00	100.00
Nutrient level, % ^4^		
Metabolic energy, kcal/kg	2660	2660
Crude protein, %	15.9	15.9
Digestible lysine, %	0.83	0.80
Digestible methionine, %	0.36	0.37
Digestible sulfur-containing amino acids, %	0.61	0.57
Digestible threonine, %	0.49	0.46
Calcium, %	3.54	3.77
Available phosphorus, %	0.38	0.36

^1^ Stone powder was added as 50% fine and 50% coarse stone powder to the experimental diet. ^2^ (FBP is the abbreviation of fish bone powder) Fish bone meal typically contains approximately 34% crude protein, 1.3% lysine, 0.9% methionine, 0.33% cysteine, 15.5% calcium, and 6% phosphorus. ^3^ DSM Company L33 premix. Each kg of feed provides the following: vitamin A9900IU, vitamin D34000IU, vitamin E25IU, vitamin K32.5 mg, vitamin B12 mg, vitamin B26 mg, vitamin B64 mg, vitamin B12 0.024 mg, biotin 0.2 mg, pantothenic acid 10 mg, nicotinamide 35 mg, folic acid 1 mg, choline 360 mg, iron 80 mg, copper 10 mg, manganese 100 mg, zinc 100 mg, iodine 1.2 mg, selenium 0.3 mg, and methionine 1.5 g. ^4^ The values in parentheses are the analyzed values of nutrient levels in the experimental basal diets. Others are calculated values.

**Table 2:** Effects of fish bone meal (FBP) supplementation on the production performance of laying hens fed a basic diet (CON).

Cycle	Item	CON	FBP	*p* Value
1–3 week	Egg weight (g)	55.56 ± 0.48	55.77 ± 0.61	0.72
	Egg production rate (%)	80.00 ± 6.23 ^a^	90.00 ± 5.55 ^b^	0.03 *
	FCR	2.18 ± 0.13	2.02 ± 0.15	0.21
4–6 week	Egg weight (g)	55.65 ± 1.05	55.77 ± 1.06	0.56
	Egg production rate (%)	96.67 ± 2.55	96.67 ± 2.44	0.98
	FCR	2.09 ± 0.13	2.03 ± 0.12	0.45
7–9 week	Egg weight (g)	59.29 ± 1.05	59.58 ± 1.03	0.77
	Egg production rate (%)	96.67 ± 2.65	98.33 ± 2.73	0.53
	FCR	1.98 ± 0.10	2.00 ± 0.12	0.72
10–12 week	Egg weight (g)	60.27 ± 1.05	60.86 ± 1.13	0.88
	Egg production rate (%)	98.33 ± 2.65	93.33 ± 3.52	0.23
	FCR	1.98 ± 0.11	2.03 ± 0.12	0.56
1–12 week	Egg weight (g)	57.69 ± 0.91	57.75 ± 0.96	0.96
	Egg production rate (%)	92.92 ± 3.27	94.58 ± 3.56	0.44
	FCR	2.06 ± 0.12	2.02 ± 0.13	0.58

^a,b^ Small letters indicate significant differences between groups (The following tables are the same) * indicates that there is a significant difference between the groups (The following tables are the same).

**Table 3:** Effects of fish bone meal (FBP) supplementation compared to the basic diet (CON) on egg quality parameters in laying hens.

Parameter	CON Group	FBP Group	*p* Value
Eggshell weight (g)	6.75 ± 0.32	7.08 ± 0.5	0.06
Eggshell thickness (mm)	0.37 ± 0.01	0.37 ± 0.02	0.31
Aspect ratio	1.31 ± 0.03	1.31 ± 0.03	0.88
Eggshell strength (kg/cm^2^)	4.37 ± 0.45	4.39 ± 0.64	0.94
Egg yolk weight (g)	17.16 ± 0.72 ^a^	18.49 ± 1.01 ^b^	<0.01 **
Yolk color	12.17 ± 0.39	12.5 ± 0.52	0.09
Protein height (mm)	9.22 ± 0.62	9.3 ± 0.82	0.79
Haugh unit	97.64 ± 2.92	97.97 ± 4.64	0.83

** indicates that there is a significant difference between the groups (The following tables are the same).

**Table 4:** Effects of fish bone meal (FBP) supplementation compared to the basic diet (CON) on intestinal morphology in laying hens.

Segment	Parameter	CON	FBP	*p* Value
Duodenum	Villus length (µm)	1083 ± 35 ^a^	1156 ± 61 ^b^	0.04 *
Duodenum	Crypt depth (µm)	139 ± 6 ^a^	152 ± 8 ^b^	0.01 *
Jejunum	Villus length (µm)	1027 ± 59	1044 ± 49	0.62
Jejunum	Crypt depth (µm)	140 ± 5	137 ± 11	0.62
Ileum	Villus length (µm)	802 ± 37	820 ± 63	0.60
Ileum	Crypt depth (µm)	121 ± 4	123 ± 6	0.46

**Table 5:** Effects of fish bone meal (FBP) supplementation compared to the basic diet (CON) on blood immune response markers in laying hens.

Indicator	CON Group	FBP Group	*p* Value
IgA ^1^	0.699 ± 0.056	0.714 ± 0.075	0.75
IgE ^2^	2.114 ± 0.185	2.151 ± 0.267	0.83
IgG ^3^	8.319 ± 0.631 ^a^	13.063 ± 1.415 ^b^	<0.01 **
IL-10 ^4^	134.645 ± 9.278 ^a^	155.491 ± 8.274 ^b^	<0.01 **
IFN-γ ^5^	89.338 ± 4.33 ^a^	94.415 ± 4.026 ^b^	<0.01 **

^1^ Immunoglobulin A, ^2^ Immunoglobulin E, ^3^ Immunoglobulin G, ^4^ Interleukin-10, ^5^ Interferon-gamma.

## Data Availability

The original contributions presented in this study are included in the article. Further inquiries can be directed to the corresponding author.
